# Effects and mechanisms of transcutaneous auricular vagus nerve stimulation on post-stroke cognitive impairment in animal studies: a systematic review and meta-analysis

**DOI:** 10.3389/fnins.2026.1935509

**Published:** 2026-09-14

**Authors:** Shuying Li, Xiaping Chen, Sihang Chen, Fu Liu, Jinlong Yin, Wenhao Hu, Jiang Xu, Xuejing Li

**Affiliations:** The Affiliated Huai'an Hospital of Xuzhou Medical University, Huai'an, China

**Keywords:** animal models, meta-analysis, neuroplasticity, post-stroke cognitive impairment, transcutaneous auricular vagus nerve stimulation

## Abstract

**Background:**

Post-stroke cognitive impairment (PSCI) substantially undermines rehabilitation adherence and poses a major obstacle to functional recovery. Transcutaneous auricular vagus nerve stimulation (taVNS), a noninvasive neuromodulation technique, has the potential to improve cognitive function, as demonstrated in various clinical and preclinical studies. Against this backdrop, we performed a meta-analysis of taVNS in experimental stroke models to quantify its cognitive benefits and probe the biological mechanisms involved in its effects.

**Methods:**

We systematically searched databases, including PubMed, The Cochrane Library, Embase, and Scopus, to identify controlled animal studies evaluating taVNS in PSCI models. Following eligibility screening, five studies were selected for final synthesis. The primary cognitive outcomes comprised escape latency in the Morris water maze and the Barnes maze. Data extraction was performed independently by two investigators, and statistical analyses were performed using Review Manager 5.4 software.

**Results:**

Five RCTs involving 158 rodents were analyzed in this study. The meta-analysis revealed that taVNS significantly improved memory function in PSCI animals (SMD = 1.85, 95% CI: 1.06 to 2.64, *p* < 0.01, I ^2^ = 57%) and learning ability (MD = − 6.38, 95% CI: − 8.87 to − 3.89, *p* < 0.01, I ^2^ = 0%) and reduced modified neurological severity scores (MD = − 1.08, 95% CI: − 1.63 to − 0.52, *p* < 0.01, I ^2^ = 38%). Mechanistically, taVNS significantly elevated acetylcholine levels (SMD = 1.57, 95% CI: 0.85 to 2.30, *p* < 0.01, I^2^ = 0%), inhibited neuronal apoptosis (SMD = 2.29), and promoted angiogenesis (SMD = 1.63). Sensitivity analysis confirmed the robustness of the primary outcomes.

**Conclusion:**

This study provides preclinical evidence confirming that taVNS can effectively improve cognitive deficits in PSCI animal models. The enhancement of Ach levels, inhibition of neuronal apoptosis, and promotion of vascular repair may be related to the non-neuronal cholinergic system (NNCS), anti-inflammatory, and antioxidant stress mechanisms. However, as only a few studies were included, more high-quality animal experiments are needed in the future to develop standardized interventions and further explore the molecular mechanisms underlying these effects.

**Systematic review registration:**

https://www.crd.york.ac.uk/prospero/display_record.php?ID=CRD420261329420.

## 1 Introduction

Stroke is an acute cerebrovascular event triggered by either sudden rupture or occlusion of a cerebral vessel, depriving the brain tissue of blood flow and ultimately leading to irreversible injuries. According to the Global Burden of Disease (GBD) 2021 study, stroke remains the third leading cause of death and the fourth leading contributor to disability-adjusted life years (DALYs) worldwide, accounting for approximately 11.9 million incident cases annually (GBD 2021 Stroke Risk Factor Collaborators, 2024). Epidemiological data indicate that the total number of stroke survivors in China reached 26 million in 2021. While advancements in medical care have bolstered post-stroke survival, the accompanying decline in quality of life has translated into a rising burden of Years of Life Lost (Ji et al., 2025). As the most populous country in the world, China has the largest number of stroke patients. Stroke is the leading cause of death and disability among Chinese adults; therefore, its incidence requires special attention (National Program for Stroke Prevention and Million Disability Reduction Initiative Expert Committee, 2025).

Among the various sequelae affecting stroke survivors, post-stroke cognitive impairment (PSCI) has become one of the most common complications that seriously impact the quality of life of patients. PSCI refers to a series of cognitive impairments that occur after a stroke, encompassing deficits in memory, attention, executive function, visuospatial processing, and language. Patients with PSCI often have impaired self-management abilities, compromising their adherence to secondary prevention medications and participation in rehabilitation training (Smith et al., 2017). Consequently, PSCI is associated with a higher risk of recurrent stroke and mortality (Dowling et al., 2024), highlighting the need for effective interventions targeting post-stroke cognitive recovery. Research has shown that cognitive impairment leads to limitations in patients’ daily activities and communication, making it difficult for them to return to work (Stolwyk et al., 2021; Cunningham et al., 2026). In addition, it is often accompanied by adverse neuropsychological outcomes, such as depression, sleep disorders, personality changes, and behavioral alterations (El Husseini et al., 2023). These sequelae, which markedly compromise daily functioning, also result in prolonged hospitalization and escalating healthcare expenditures (Huang et al., 2022).

The current clinical management of PSCI is anchored in the principle of “early screening and timely intervention,” focusing on risk factor modification, pharmacotherapy, and rehabilitation. No pharmacological agent has been proven to fully reverse the pathological trajectory of PSCI. Existing treatment options, including cholinesterase inhibitors, offer only modest efficacy and are frequently accompanied by nausea, insomnia, and other adverse effects, undermining long-term treatment adherence (Kreiger et al., 2025; Ravi et al., 2025). Therefore, it is particularly urgent to explore effective interventions for PSCI. Finding a safe and effective treatment is of great significance for improving patient outcomes and alleviating socioeconomic burdens. In response to this need, neuromodulation technologies, such as vagus nerve stimulation, have gained public attention.

As the main component of the parasympathetic nervous system, the afferent fibers of the vagus nerve terminate in the nucleus of the solitary tract, which projects directly or indirectly to the locus coeruleus and activates the noradrenergic system. The norepinephrine (NE) released in turn can affect the lateral amygdala (AMY), hippocampus (HC), and cortical regions (such as the medial prefrontal cortex), thereby regulating memory formation and enhancing neural plasticity. This anatomical pathway constitutes the neural basis for the regulation of cognitive function (Berthoud and Neuhuber, 2000; Colzato and Beste, 2020; Hilz, 2022; Szeska et al., 2025). Building on this foundation, vagus nerve stimulation (VNS), a technique that modulates neural activity through electrical stimulation, has been widely applied in the field of neuropsychiatric disorders (Johnson and Wilson, 2018). Early VNS required surgical implantation of electrodes into the cervical vagus nerve trunk, that is, invasive vagus nerve stimulation (invasive VNS, iVNS). Transcutaneous vagus nerve stimulation (tVNS) has emerged as a promising alternative to invasive stimulation. TVNS stimulates the superficial branch of the vagus nerve via cutaneous surface electrodes, avoiding surgical trauma and offering safety and compliance. According to the stimulation site, tVNS can be further divided into transcutaneous cervical VNS (tcVNS) and transcutaneous auricular VNS (taVNS) (Hilz, 2022). TaVNS exerts its effects by electrically stimulating the auricular branch of the vagus nerve (ABVN) and has been shown to facilitate functional recovery through multiple mechanisms, including suppression of neuroinflammation, preservation of blood–brain barrier integrity, and promotion of angiogenesis (Liu et al., 2024). Compared with iVNS, taVNS offers distinct advantages: its entirely noninvasive design not only bolsters patient adherence but also eliminates the risks associated with surgical implantation and anesthesia, positioning it as a safer, more cost-effective, and inherently more sustainable strategy for long-term neuromodulation. Notably, a recent Cochrane systematic review evaluated invasive and non-invasive VNS combined with rehabilitation versus rehabilitation alone for upper-limb motor recovery after stroke; although the certainty of the evidence was rated very low to low, VNS appeared to confer modest short-term benefits without substantially increasing adverse events (Everard et al., 2026).

Evidence linking taVNS to cognitive enhancement has thus far been drawn predominantly from healthy cohorts and Alzheimer’s disease models. However, direct clinical evidence is emerging. In a recent randomized controlled trial of 90 people with chronic stroke, 6 weeks of transcutaneous vagus nerve stimulation delivered concurrently with upper-limb exercise significantly improved cognitive function (Montreal Cognitive Assessment) and upper-limb motor function compared with sham stimulation, and the cognitive benefits persisted at a one-month follow-up (Chan and Ng, 2026). Nevertheless, such clinical trials remain scarce, target primarily chronic-stage patients with heterogeneous cognitive outcomes, and provide limited insight into the underlying mechanisms. Preclinical investigations specifically targeting PSCI remain fragmented, and systematic appraisals of treatment efficacy are conspicuously absent. Animal models serve as indispensable platforms for screening therapeutic targets and dissecting molecular mechanisms, thereby holding decisive weight in determining the feasibility of the clinical translation of research findings. The primary objective of this study was to systematically review and meta-analyze the available preclinical evidence regarding the efficacy of taVNS in improving cognitive performance in animal models of ischemic stroke-induced cognitive impairment, to investigate its possible mechanisms of action, and to determine whether these effects are distinguishable from those observed with sham stimulation or the absence of stimulation. Thereby addressing current gaps in the field and providing a comprehensive evidence base for clinical translation.

## 2 Methods

### 2.1 Review protocol and registration

This study was conducted in accordance with the guidelines of the Preferred Reporting Items for Systematic Reviews and Meta-Analyses (PRISMA) (Page et al., 2020) and Cochrane Handbook. The study protocol was prospectively registered in the PROSPERO database (registration number: CRD420261329420) to ensure transparency and to minimize the risk of bias. Any deviations from the original protocol were documented in the final report.

### 2.2 Literature search strategy

This study systematically searched four English-language databases: PubMed, The Cochrane Library, Embase, and Scopus. The search covered each database from inception to March 2, 2026. For studies whose full texts were not accessible via electronic databases, we contacted the corresponding authors to request the manuscript or sought alternative acquisition routes. No supplementary searches, including handsearching, reference list examination, or trial registry consultation, were conducted; the electronic database search was considered sufficient to capture the eligible literature. The search strategy was based on the PICO (Schardt et al., 2007) framework and incorporated both MeSH terms and free-text keywords. Search terms included: (1) transcutaneous auricular vagus nerve stimulation (e.g., “taVNS,” “transcutaneous auricular vagus nerve stimulation”, “Nerve Stimulations, Vagus”); and (2) post-stroke cognitive impairment (e.g., “post-stroke cognitive impairment”, “cerebrovascular accident”, “stroke”). There were no restrictions on language or publication year. The detailed search steps are provided in Supplementary material 1.

### 2.3 Inclusion and exclusion criteria

This study followed the PICOS guidelines for article selection and inclusion criteria. Inclusion criteria were as follows: (1) Study design: randomized controlled trial design. (2) Subjects: studies included animal models of stroke (including ischemic stroke, hemorrhagic stroke/ICH) or traumatic brain injury, regardless of sex, age, or species; (3) Intervention: the experimental group received non-invasive (taVNS) therapeutic intervention. The control group consisted of stroke or brain injury animal models that did not receive active taVNS intervention or received sham stimulation; (4) Outcomes: studies were required to report quantitative data (such as mean, standard deviation, and sample size) for at least one of the following outcomes: the primary outcome was behavioral testing assessing learning and memory ability, such as escape latency and platform-crossing number in the Morris water maze (MWM); secondary outcomes included modified neurological severity score (mNSS) or neural cell recovery parameters (such as neuronal count and apoptosis level); and (5) studies published in full-text form in peer-reviewed journals.

The exclusion criteria were as follows: (1) no taVNS intervention or combination with other treatments; (2) not using rodent models or animal models without cognitive impairment; (3) absence of the specified control group in experimental studies; (4) studies not reporting the required behavioral or neuropathological outcomes; (5) *in vitro* studies, clinical trials, case reports, abstracts, duplicate data, reviews, and incomplete texts; (6) unclear sample size; and (7) non-English articles for which a complete translation was not obtainable.

### 2.4 Data extraction and management

Retrieved records were managed using the EndNote 21 software. After deduplication, two researchers independently assessed the deduplicated literature according to the inclusion and exclusion criteria, first reviewing the titles and abstracts of relevant papers, followed by an examination of the full texts of these studies. Any discrepancies between the two independent reviewers were initially settled by discussion. In cases where consensus could not be achieved, a third reviewer arbitrated the final decision.

Studies meeting the eligibility criteria proceeded to the data extraction phase. Data extraction was performed using Excel spreadsheets, with extracted information divided into three sections: (1) Basic parameters, including publication year, first author, animal species, sex, body weight, sample size, and animal model type; (2) Interventions, detailing the stimulation frequency, current intensity, daily intervention duration, and total treatment period of taVNS; and (3) Outcomes, including behavioral data on learning and memory ability, mNSS score, and neural cell recovery indicators. If a study included multiple independent experimental groups, each experimental group and its corresponding control group were considered for independent comparison. When outcome data were presented only in graphical form, numerical data were extracted using WebPlotDigitizer software. The two reviewers performed the extraction independently and then cross-checked all extracted results to ensure accuracy.

### 2.5 Quality assessment

We used the SYRCLE risk of bias tool (Hooijmans et al., 2014), which was specifically designed for animal studies, to assess the quality of the included studies. This tool is structured around 10 key domains intended to cover potential sources of selection, performance, detection, attrition, reporting, and other biases. The ten domains assessed are: (1) Sequence generation, (2) Baseline characteristics, (3) Allocation concealment, (4) Random housing, (5) Blinding of investigators, (6) Random outcome assessment, (7) Blinding of outcome assessors, (8) Incomplete outcome data, (9) Selective outcome reporting, and (10) Other sources of bias. Based on the information provided in the original articles, each item was classified as “low risk of bias (yes),” “high risk of bias (no),” or “unclear risk of bias (unclear).” Any disagreements between the two researchers were resolved through in-depth discussions or by consulting a third expert. Disagreements on methodological details were resolved through discussion between the two reviewers, whereas fundamental disagreements regarding the interpretation of the results were adjudicated by a third reviewer. A sensitivity analysis was performed to assess the impact of individual studies on the overall results. However, due to the limited number of included studies, no formal sensitivity analysis was conducted. Instead, the exclusion method was used individually when heterogeneity was high.

The certainty of evidence for each outcome was assessed by two reviewers using the GRADE framework (Guyatt et al., 2008), which was adapted for preclinical animal studies based on the domains of risk of bias, inconsistency, indirectness, imprecision, and publication bias.

### 2.6 Statistical analysis and heterogeneity assessment

Meta-analysis was conducted using Review Manager 5.4 software. For outcome measures assessed using different evaluation methods or instruments, the standardized mean difference (SMD) was chosen as the effect size; for those assessed using identical methods, the mean difference (MD) was selected. According to Cohen’s criteria (Cohen, 1988), SMD values of 0.2, 0.5, and 0.8 were interpreted as small, moderate, and large effects, respectively. Heterogeneity among studies was assessed using the I^2^ statistic and the Q test: when I^2^ ≤ 50% and *p* > 0.1, heterogeneity was considered low and a fixed effects model was used; if I^2^ > 50% or *p* < 0.1, this was considered significant heterogeneity and a random effects model was applied. In the case of high heterogeneity, the source of heterogeneity was explored, and the stability of the results was tested by sequentially excluding studies. Statistical significance was defined as *p* < 0.05. Effect sizes were calculated based on the post-intervention means and standard deviations for each group. For studies with multiple arms, each experimental (taVNS) group was compared with its corresponding control group; when both sham stimulation and no-stimulation control groups were available, the sham stimulation group was preferentially selected as the comparator.

## 3 Results

### 3.1 Literature selection process (PRISMA flow diagram)

Through systematic searches of four electronic databases—PubMed (*n* = 34), The Cochrane Library (*n* = 57), Embase (*n* = 98), and Scopus (*n* = 2,021)—a total of 2,210 relevant articles were initially identified. After removing 43 duplicates using Endnote 21, the remaining 2,167 studies were preliminarily screened by title and abstract. At this stage, 2,111 articles were excluded for not meeting the prespecified inclusion criteria. Subsequently, two researchers independently reviewed the full texts of the remaining 56 studies for inclusion. Following detailed assessment, 51 studies were further excluded for the following reasons: wrong intervention (*n* = 2), wrong population (*n* = 17), uncompleted clinical trials (*n* = 30), and insufficient data available (*n* = 2). Ultimately, five studies meeting the criteria were included in this systematic review and meta-analysis. The literature search and selection processes are detailed in Figure 1.

**Figure 1:**
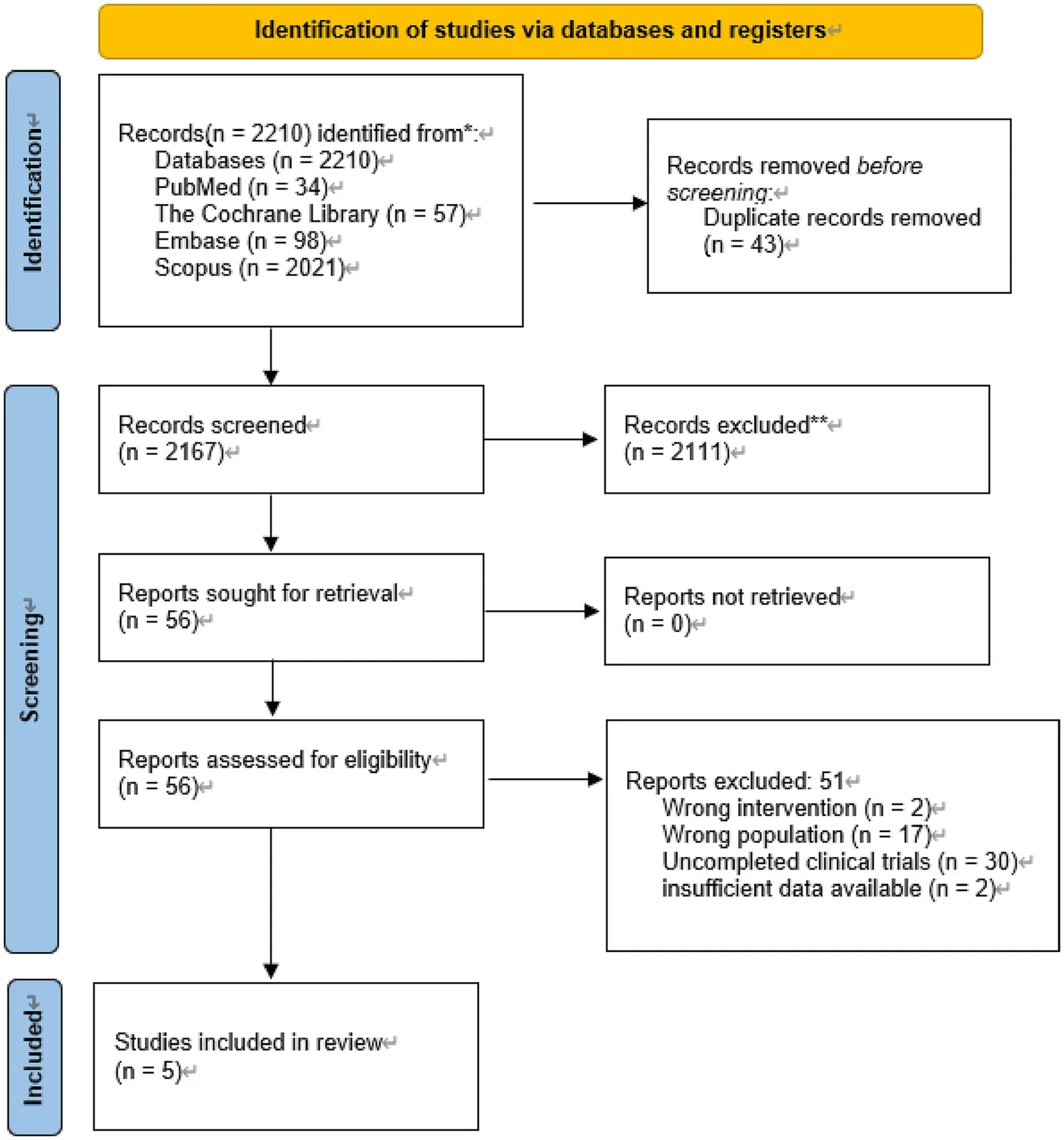
PRISMA flow diagram summarizing the study selection process.

### 3.2 Characteristics of included studies

All the included studies were published between 2016 and 2025. The animal models used in the included studies were rats or mice, with three studies utilizing rats (Kuo et al., 2016; Wu et al., 2018; Sun R. et al., 2025) and two utilizing mice (Choi et al., 2022; Gong et al., 2025). In three studies, the animal models were 8 weeks old (Wu et al., 2018; Choi et al., 2022; Gong et al., 2025), in one study, they were 6 weeks old (Sun R. et al., 2025), and one study did not specify the age of the animals (Kuo et al., 2016). All five studies involved adult male animal models. Regarding intervention characteristics, except for one study in which the stimulation site was bilateral ears (Kuo et al., 2016), all others targeted the left ear. The number of intervention sessions in each study ranged from 5 to 14, with a duration of 20–60 min. The frequency of taVNS in three studies (Sun R. et al., 2025; Choi et al., 2022; Gong et al., 2025) was 20 Hz, in one study (Wu et al., 2018) was 15 Hz, and in one study (Kuo et al., 2016) was 2 Hz. The intensity of taVNS was reported as 2 mA in one study (Kuo et al., 2016), 1 mA in three studies (Sun R. et al., 2025; Choi et al., 2022; Gong et al., 2025), and 0.8 mA in one study (Wu et al., 2018). One study (Kuo et al., 2016) used a pulse width of 100 μs, two studies (Sun R. et al., 2025; Choi et al., 2022) used 330 μs, one study (Gong et al., 2025) used a pulse width of 330 ms, and another study (Wu et al., 2018) did not report the pulse duration. The devices used in the five studies differed. The detailed information of these five included studies is shown below in Tables 1, 2.

**Table 1:** Main characteristics of animals.

Author	Year	Country	Species of model	Ischemic			Animal species	Age	Weight
				Stroke model	Time (min)	Side			
Kuo et al. (2016)	2016	China	Rat	tMCAO	10	Right	Male SD rats	NA	280–310 g
Wu et al. (2018)	2018	China	Rat	tMCAO	60	Left	Male SD rats	8-week-old	200–220 g
Choi et al. (2022)	2022	Korea	Mice	tBCCAO	25	Bilateral	Male C57BL/6 mice	8-week-old	NA
Gong et al. (2025)	2025	China	Mice	tMCAO	60	Left	Male C57BL/6 J mice	8-week-old~9-week-old	22–25 g
Sun R. et al. (2025)	2025	China	Rat	tBCCAO	25	Bilateral	SPF Male SD rats	6-week-old	250 g

tMCAO, transient middle cerebral artery occlusion; tBCCAO, transient bilateral common carotid artery occlusion; SPF, specific-pathogen-free; SD, Sprague–Dawley.

**Table 2:** Characteristics of intervention methods and outcomes.

Author	Intervention								Outcome
	Intervention methods	Stimulation site	Intervention count	Intervention duration (min)	Interventional instrument	Stimulation frequency	Stimulation intensity	Stimulation pulse width	
Kuo et al. (2016)	ES	Ears (cathode in the apex and anode in the lobe)	7	20/day (10 for each ear)	ES apparatus (Trio 300, Ito, Japan)	2 Hz	2 mA	100 μs	Neurological deficit score; STL in the passive avoidance test; rotarod test times; increase nAChR α4 release/could not change Ki67 protein levels but could increase astrocytes and neuronal cells
Wu et al. (2018)	tVNS	The cavum conchae and cymba concha of the left ear respectively	5	60	Two acupuncture needles (Hwatuo, Suzhou, China);an electrical device by wires (Hans electrical stimulation device, Nanjing, China)	15 Hz	0.8 mA	NA	mNSS; MWM; Infarct volume; enhanced the Ach release (The expression of mRNA of ChAT, CarAT)
Choi et al. (2022)	taVNS	The left ear. Stimulate the cymba concha and the surface of the dorsal auricle where ABVN innervates.	The long-term treatment group received 6 days of treatment and was tested on day 7; the short-term treatment group received 2 days of treatment and was tested 3 days after tBCCAO surgery.	20	Developed a novel taVNS device	20 Hz	1 mA	330 μs	The discrimination index scores determined by the NOR test; the rates of spontaneous alternations in the Y-maze test; Promot CSF Circulation
Gong et al. (2025)	atVNS	At the left ear’s auricle	5	30	YC-3 Bipolar Programmable Stimulator, Chengdu Instruments Factory, Chengdu, China	20 Hz	1 mA	330 ms	mNSS; Barnes Maze; Infarct volume; Promote Neurogenesis and Angiogenesis
Sun R. et al. (2025)	taVNS	The left ear; the cymba conchae and the dorsal auricular surface	14	30	The therapeutic setup consisted of three components: electrode sheets, lead wires, and a Huatuo-brand electroacupuncture instrument.	20 Hz	1 mA	330 μs	MWM; NOR; taVNS enhances the expression of the TJ proteins and VEGF by activating the Wnt7/β-catenin signaling pathway, thereby reducing BBB leakage and promoting angiogenesis.

ES, electric stimulation; tVNS, transcutaneous vagus nerve stimulation; taVNS, transcutaneous auricular vagus nerve stimulation; atVNS, auricular vagus nerve stimulation; ABVN, auricular branch of the vagus nerve; CSF, cerebrospinal fluid; BBB, blood–brain barrier; MWM, Morris Water Maze; mNSS, modified neurological severity score; NOR, novel object recognition; ChAT, choline acetyltransferase; CarAT, carnitine acetyltransferase; TJ, tight junction; VEGF, vascular endothelial growth factor.

### 3.3 Methodological quality of included studies

The methodological quality of the five included animal studies was appraised with the SYRCLE’s Risk of Bias tool. Only one study adequately described the method of sequence generation, and two provided sufficient details on allocation concealment. Blinding of caregivers and/or investigators emerged as the predominant methodological weakness—none of the studies offered a comprehensive account of the blinding procedures employed. All studies were rated as “low risk” for both baseline characteristics and random housing (the living environment of experimental animals), underscoring the strong baseline comparability between the experimental and control groups. The blinding of outcome assessors was not addressed in two studies. The remaining three participants described the procedures used. Reassuringly, all studies reported complete outcome data. Random outcome assessment and selective outcome reporting were judged to be at low risk of bias across all the included studies, as each followed a prespecified protocol and documented the methods of outcome measurement in detail. The full bias assessment is presented in Figure 2.

**Figure 2:**
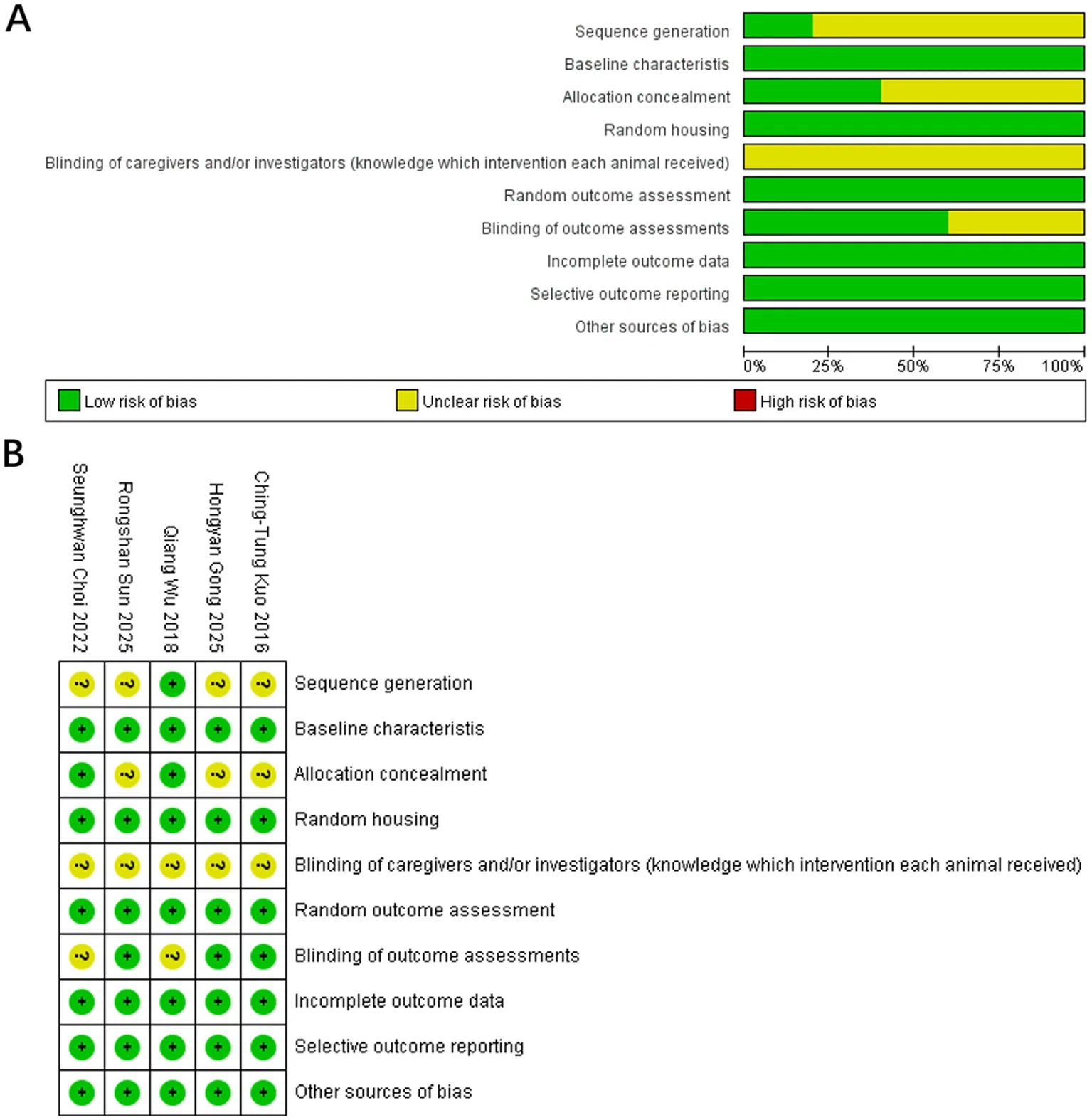
Risk of bias assessment results for five studies based on SYRCLE’s risk of bias tool. **(A)** Risk of bias graph; **(B)** Risk of bias summary.

### 3.4 Meta-analysis of primary outcomes: learning and memory

#### 3.4.1 Primary outcome—memory

The included studies employed a battery of behavioral tests to probe cognitive recovery in stroke animal models to capture both spatial learning and memory, such as the passive avoidance test, Morris water maze (MWM), Barnes maze, novel object recognition (NOR) test, and Y-maze tests. Memory performance was indexed by step-through latency, time spent in the target quadrant, platform crossing frequency, and rates of spontaneous alternation. All five studies reported the effects of taVNS on spatial memory in stroke animal models, involving a total of 98 animals. The overall results showed that taVNS significantly improved stroke-induced spatial memory impairment (SMD: 1.85 [95% CI: 1.06, 2.64], *p* < 0.01, I2 = 57%, Figure 3A). Based on the GRADE framework, the certainty of evidence for this outcome was rated low, primarily owing to methodological limitations in blinding, considerable heterogeneity (I^2^ = 57%), and the small number of animals analyzed.

**Figure 3:**
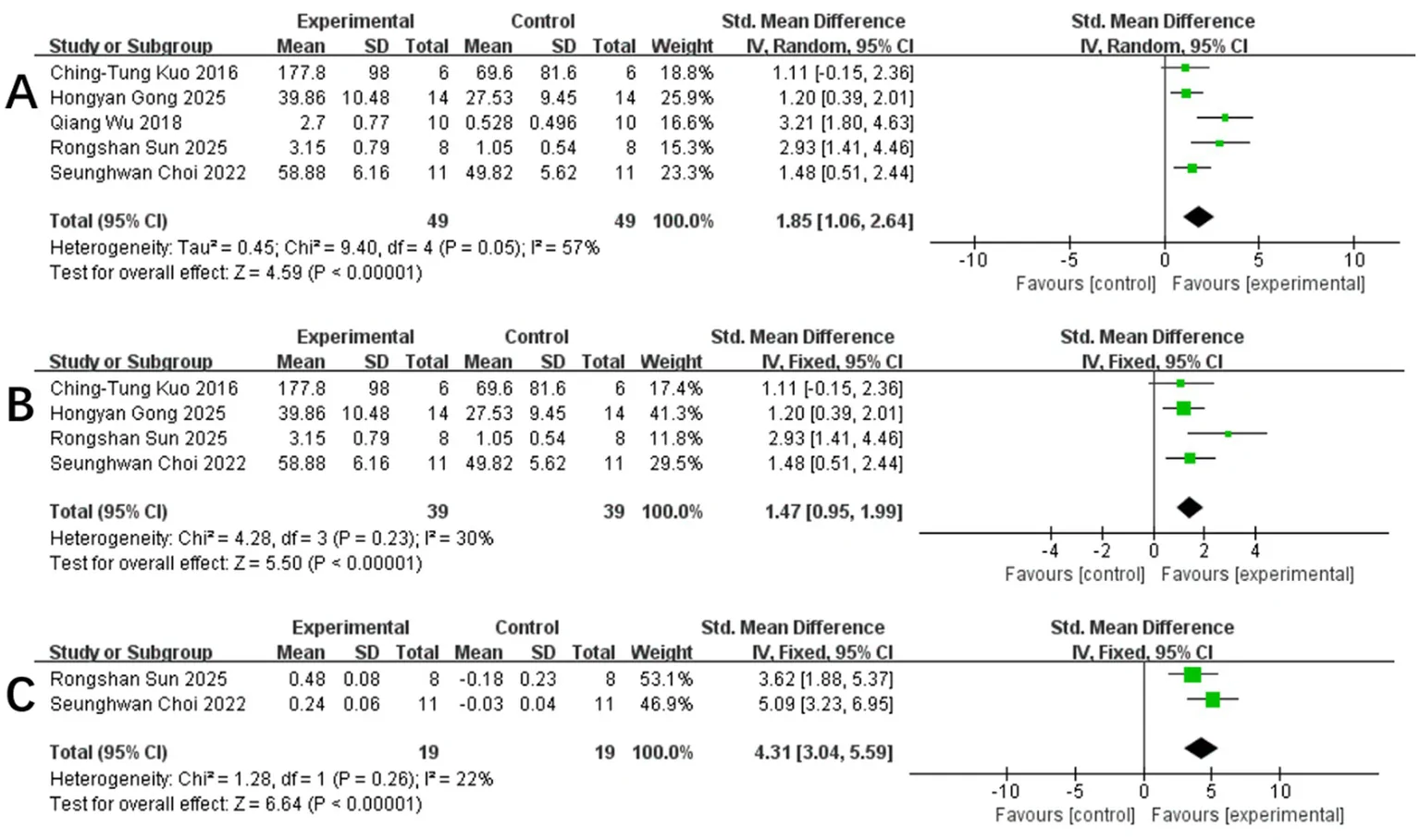
The forest plot shows the effect of taVNS on memory function. **(A)** All five studies were included; **(B)** Results after sensitivity analysis; **(C)** Effect of taVNS on discrimination index scores from the NOR test. Data are presented as standardized mean differences (SMD) with 95% confidence intervals (CI).

The above analysis results indicate a moderate degree of heterogeneity in the studies. Owing to the limited number of studies included, we conducted a sensitivity analysis using a leave-one-out approach. The results showed that Wu et al. (2018) were the main source of heterogeneity. After excluding this study, the heterogeneity dropped to 30%, but the pooled effect size still indicated that taVNS had a statistically significant effect on memory improvement (SMD: 1.47 [95% CI: 0.95, 1.99], *p* < 0.01, I2 = 30%, Figure 3B). Upon full-text review, we believe that the effect size is robust but is influenced by one study with a longer stimulation duration. Nevertheless, the consistency of the sensitivity analysis results suggests that the effect of taVNS on improving memory function in animal models of post-stroke cognitive impairment is robust. Therefore, we chose not to exclude this study from the final results. To assess publication bias, we generated a funnel plot (Figure 4). The distribution of the scatter points was symmetrical, suggesting a low likelihood of publication bias.

**Figure 4:**
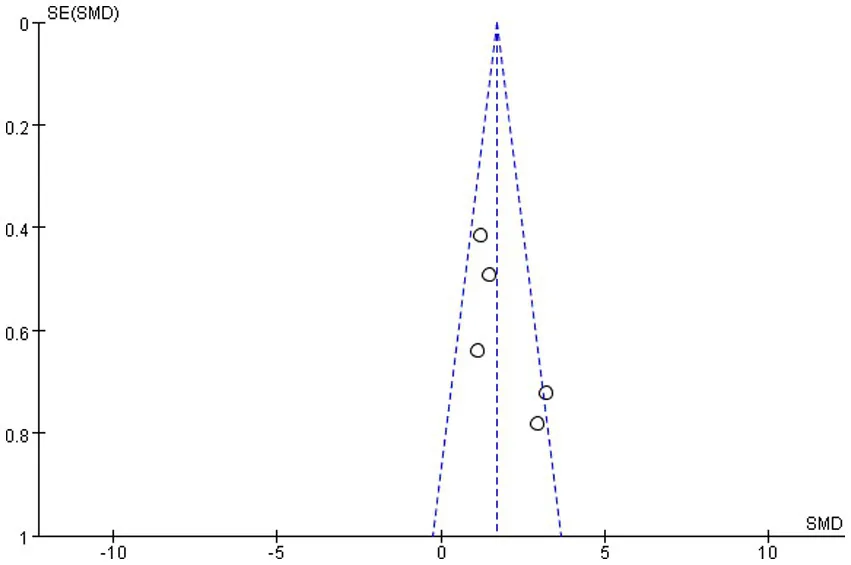
Funnel plots illustrating the presence of publication bias.

As a test that assesses changes in memory ability in animals by utilizing rodents’ spontaneous behavior rather than post-training behavior, the NOR test has the advantage of requiring less time and fewer procedures, thereby minimizing the risk of bias (Ennaceur and Delacour, 1988). Two studies (Sun R. et al., 2025; Choi et al., 2022) used discrimination index scores from the NOR to assess recognition memory in stroke animal models. The assessment results were consistent with those of the other memory-related tests mentioned above, all indicating that taVNS significantly improved recognition memory abilities in stroke animal models (SMD: 4.31 [95% CI: 3.04, 5.59], *p* < 0.01, I2 = 22%, Figure 3C). Based on the GRADE framework, the certainty of evidence was rated low and downgraded for risk of bias and imprecision owing to the small number of studies.

#### 3.4.2 Primary outcome—learning

Learning ability included in the study was uniformly assessed by measuring escape latency. Only three studies (Wu et al., 2018; Sun R. et al., 2025; Gong et al., 2025) reported relevant data, among which one study by Gong et al. (2025) used the Barnes maze, while the other two used the MWM. The results showed that taVNS produced a statistically significant improvement in stroke-induced decline in learning ability (MD − 6.38, 95% CI − 8.87 to −3.89; *P* < 0.01; I^2^ = 0%; Figure 5). Based on the GRADE framework, the certainty of evidence was rated as moderate and downgraded for risk of bias, but with consistent effect estimates across studies (I^2^ = 0%).

**Figure 5:**
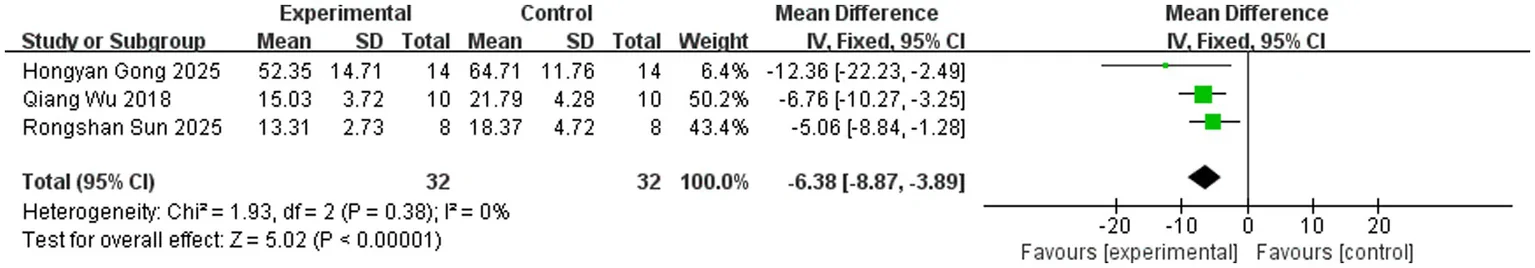
The forest plot shows the effect of taVNS on learning ability. Data are presented as mean differences (MD) with 95% confidence intervals (CI).

#### 3.4.3 Secondary outcome—modified neurological severity score

Neurological deficit scores are commonly used to evaluate the severity of neurological impairment in animal stroke models, and three studies (Kuo et al., 2016; Wu et al., 2018; Gong et al., 2025) assessed the effect of taVNS on neurological deficit scores. Pooled effect estimates indicated that taVNS improved neurological deficit scores in animal stroke models (MD: −1.08 [95% CI: −1.63, −0.52], *p* < 0.01, I2 = 38%, Figure 6). Based on the GRADE framework, the certainty of evidence was rated as low, downgraded for risk of bias and imprecision.

**Figure 6:**
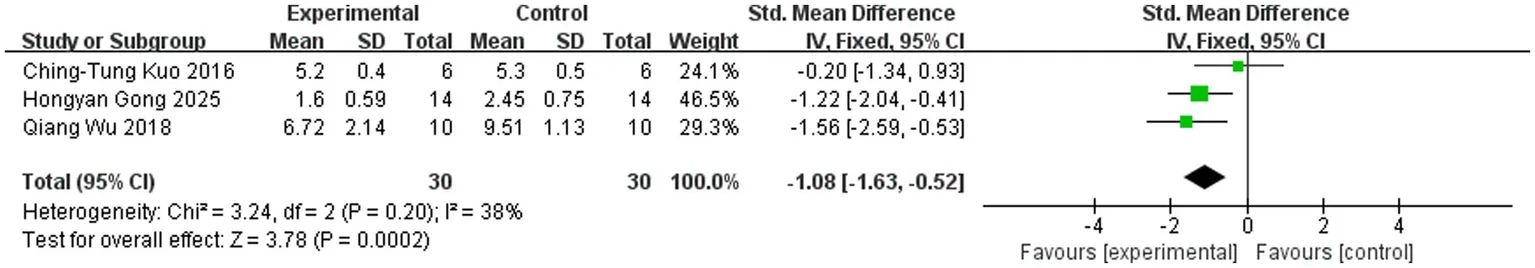
The forest plot shows the effect of taVNS on the modified neurological severity score. Data are presented as mean differences (MD) with 95% confidence intervals (CI).

Beyond the mNSS, we examined whether the included studies reported sensorimotor outcomes separately from cognitive measures. Three studies (Kuo et al., 2016; Wu et al., 2018; Gong et al., 2025) assessed sensorimotor function as part of the mNSS or through dedicated neurological scores. Notably, Wu et al. explicitly reported that taVNS reversed post-ischemic limb dysfunction, including forelimb movement disorder, in addition to improving Morris water maze performance (Wu et al., 2018). The remaining two studies (Sun R. et al., 2025; Choi et al., 2022) focused on cognitive and vascular pathological outcomes and did not report independent sensorimotor assessment. Thus, sensorimotor recovery was documented in the majority of the pooled studies, but its contribution to the observed cognitive improvements could not be separated quantitatively.

#### 3.4.4 Secondary outcome—infarct volume

Infarct volume, which serves as an indicator of lesion size, can represent the extent of irreversible damage. Two of the included studies reported data on this outcome. One study by Wu et al. (2018) reported that taVNS has a positive effect on limiting infarct size. Another study by Gong et al. (2025) showed that although there was numerical improvement, there was no statistically significant difference between the taVNS group and the control group with a relatively small sample size.

#### 3.4.5 Neuropathological features

The included studies examined pre- and post-experiment acetylcholine (Ach) and its receptor levels, changes in the number of neuron-associated cells in cortical and hippocampal regions, glial fibrillary acidic protein (GFAP)-positive stained cells, increases in angiogenesis-related factors, and mean flow speed of microspheres to explore the neuropathological mechanisms by which taVNS improves cognitive impairment after ischemic stroke.

Three studies (Kuo et al., 2016; Wu et al., 2018; Gong et al., 2025) examined changes in acetylcholine (ACh) levels and receptor expression. Compared to the controls, taVNS-treated animals exhibited elevated ACh concentrations and upregulated receptor expression (SMD: 1.57 [95% CI: 0.85, 2.30], *p* < 0.01, I2 = 0%, Figure 7A). Three studies (Kuo et al., 2016; Sun R. et al., 2025; Gong et al., 2025) quantified the changes in neuronal cell populations following taVNS intervention in stroke models. The pooled data indicated that taVNS significantly attenuated post-stroke neuronal apoptosis (SMD: 2.29 [95% CI: 1.17, 3.40], *p* < 0.01, I2 = 0%, Figure 7B). Three studies (Kuo et al., 2016; Sun R. et al., 2025; Gong et al., 2025) investigated astrocyte changes in the brain. Among them, Kuo et al. (2016) found that in the SO, SR, and DG regions of the hippocampus, the number of GFAP-positive stained cells was higher in the taVNS treatment group than in the control group. Another study by Gong et al. (2025) reported that in both the peri-infarct cortex and hippocampal CA1 regions, taVNS treatment effectively inhibited proliferation of astrocytes. Sun R. et al. (2025) demonstrated that taVNS treatment restored the balance between A1 and A2 reactive astrocyte phenotypes in post-stroke animal models. In addition, two studies (Sun R. et al., 2025; Gong et al., 2025) reported a significant increase in angiogenesis following taVNS treatment (SMD: 1.63 [95% CI: 0.32, 2.95], *p* < 0.01, I2 = 0%, Figure 7C). Only one study mentioned the mean flow speed of microspheres, indicating that taVNS treatment enhanced cerebrospinal fluid (CSF) flow in the perivascular space. Based on the GRADE framework, the certainty of evidence for these neuropathological outcomes was rated low because the mechanistic endpoints were derived from a small number of studies and represented indirect evidence of cognitive benefit.

**Figure 7:**
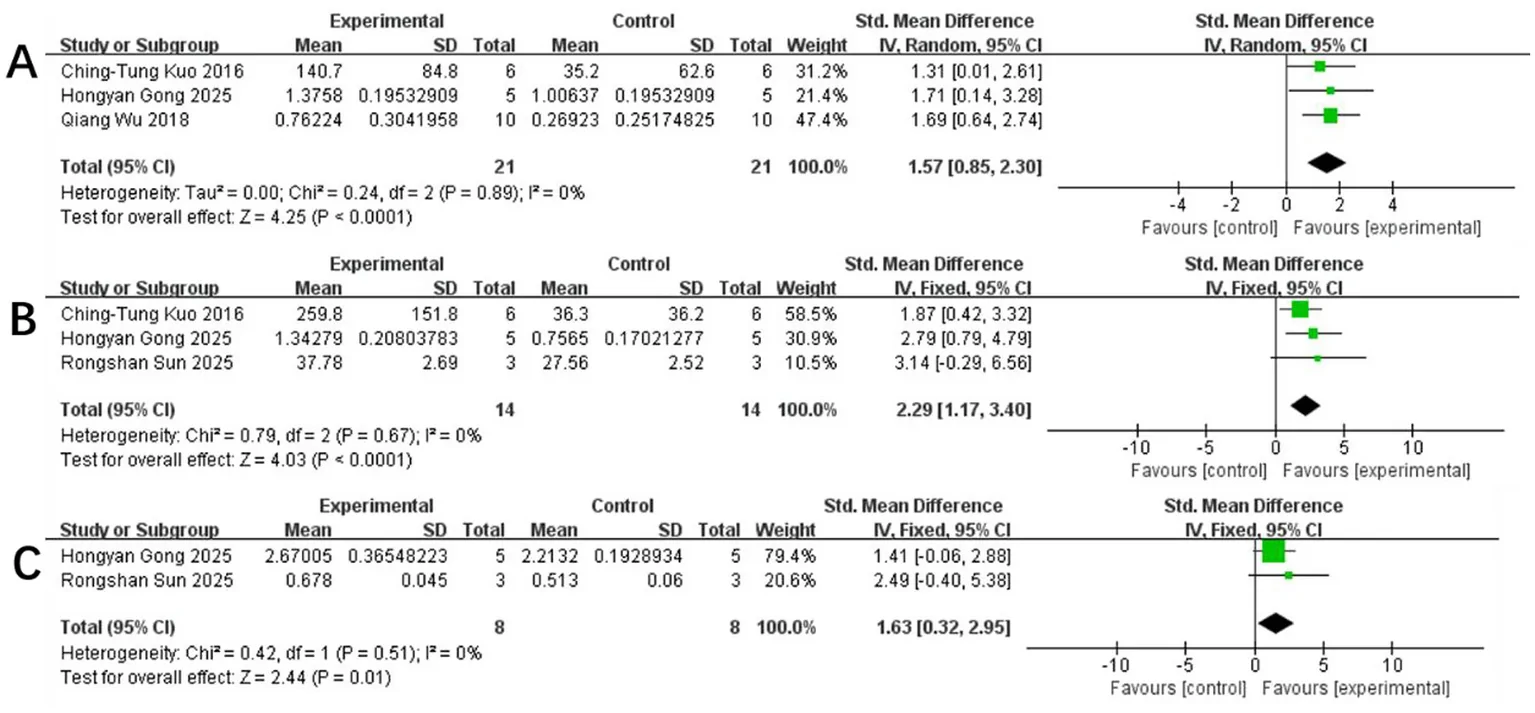
The forest plot shows the effects of taVNS on neuropathological features. **(A)** The impact on acetylcholine (ACh) levels and receptor expression; **(B)** The effect on neuronal cells; **(C)** The influence on epidermal angiogenesis. Data are presented as standardized mean differences (SMD) with 95% confidence intervals (CI).

#### 3.4.6 GRADE assessment of the certainty of evidence

We applied the GRADE framework to rate the certainty of the evidence for each pooled outcome. Because all included studies were preclinical and exhibited incomplete reporting of blinding and allocation concealment, and because the analyses were based on a small number of studies and animals, the certainty of evidence was rated low to very low for most outcomes. Specifically, the certainty of evidence was rated moderate for learning ability (escape latency), low for spatial memory, recognition memory (NOR), mNSS, and neuropathological outcomes (ACh, neuronal apoptosis, and angiogenesis), and very low for infarct volume, where the two available studies reported inconsistent findings and no meta-analysis was performed. These ratings should be considered when interpreting the therapeutic effects of taVNS.

## 4 Discussion

TaVNS has been shown to improve cognitive function in healthy adults, particularly in attention and working memory (Ridgewell et al., 2021). However, its therapeutic potential in the context of PSCI has not been systematically examined. The present synthesis of available preclinical evidence reveals that taVNS confers substantial therapeutic benefits in animal models of PSCI. Learning ability, as indexed by escape latency in the Morris water and Barnes mazes, was consistently improved by taVNS. Memory function, encompassing both spatial and recognition domains, was also significantly enhanced. Neurological severity, as reflected by mNSS scores, was reduced by taVNS, although robust evidence for infarct volume reduction remains lacking, a gap that future preclinical studies should strive to address. Collectively, these findings indicate the consistent cognitive and neuroprotective benefits of taVNS in PSCI models.

When interpreting the cognitive benefits reported here, some caution is still needed because the assessment of cognition may be affected by sensorimotor recovery, and the results may not reflect cognition alone. Most behavioral outcomes in this meta-analysis require intact swimming and locomotor ability, such as escape latency in the Morris water maze and Barnes maze, and several included studies also observed that taVNS improved limb function along with cognitive gains (Wu et al., 2018). In experimental stroke, poor water maze performance may reflect sensory and motor impairment, not necessarily a pure memory deficit. Bingham et al. used rats with middle cerebral artery occlusion to show that poor water maze performance was mainly caused by sensory and motor impairments, not a true spatial memory deficit, and they recommended probe trials and training to a preset performance criterion to separate the two (Bingham et al., 2012). These findings highlight the importance of sensorimotor confounding in stroke behavioral assessment, although their model setting was not fully consistent with the studies included in this meta-analysis. Callaghan et al. also emphasized this point, pointing out that most experimental stroke studies rely on sensorimotor testing, that non-sensorimotor outcomes are assessed in only a minority of studies, and that methodological confounding is common in widely used behavioral tests (Callaghan et al., 2025). Given these considerations, the apparent cognitive improvements observed here may partly come from improved sensorimotor function rather than a separate memory effect. Future studies are advised to include standardized sensorimotor assessments and cognitive tests with lower motor demands, and where possible, to report cognitive outcomes after sensorimotor function has stabilized.

Extrapolating these preclinical findings to the clinical management of PSCI is an enticing prospect that demands rigorous validation in patient populations. Although our data are confined to rodent models, they offer meaningful insights for future translational efforts. Invasive VNS (iVNS) was introduced as a surgical treatment for epilepsy and subsequently gained a foothold in depression, migraine, and other disorders (Bottomley et al., 2019; Panebianco et al., 2022; Yuan et al., 2025). In recent years, a growing body of evidence has suggested that vagus nerve stimulation may hold potential for treating cognitive impairment in Alzheimer’s disease, although the current clinical evidence remains largely preliminary and is derived mainly from small pilot studies (Sjogren et al., 2002; Merrill et al., 2006) and a recent scoping review of clinical and preclinical studies (Kamoga et al., 2024). As a noninvasive alternative, taVNS inherits the neurorestorative potential of traditional VNS, and owing to its non-requirement for surgical implantation and high safety profile, taVNS has attracted wide attention in the treatment of neurological disorders accompanied by cognitive impairment (Jost et al., 2026). Notably, a recent randomized controlled trial in 90 people with chronic stroke reported that transcutaneous vagus nerve stimulation delivered concurrently with upper-limb exercise significantly improved cognitive and upper-limb motor function relative to sham stimulation, offering preliminary clinical evidence for the translational promise of taVNS (Chan and Ng, 2026), although the trial enrolled chronic-stage patients and assessed cognition alongside motor outcomes, underscoring the need for dedicated trials specifically targeting PSCI. Therefore, the positive effects observed in animal models provide a theoretical basis for further clinical trials of taVNS in patients with PSCI.

Another consideration is the relationship between the side of the ischemic lesion and the side of taVNS application. The five included studies varied considerably in this regard: two studies used left-sided MCAO with ipsilateral (left-ear) stimulation (Wu et al., 2018; Gong et al., 2025), one study used right-sided MCAO with bilateral auricular stimulation (Kuo et al., 2016), and two studies induced bilateral cerebral ischemia (tBCCAO) while applying taVNS to the left ear (Sun R. et al., 2025; Choi et al., 2022). Importantly, none of the original studies explicitly reported whether the efficacy of taVNS depended on the laterality of the lesion relative to the stimulated ear, and no subgroup or sensitivity analyses addressing this question were possible in our meta-analysis. Because auricular vagal afferents project bilaterally to the nucleus of the solitary tract and thereby engage the locus coeruleus and hippocampus regardless of the side of stimulation, efficacy should not require ipsilateral placement (Berthoud and Neuhuber, 2000). Nevertheless, whether lesion-side and stimulation-side matching influences the magnitude of cognitive benefits remains an open empirical question. Therefore, we recommend that future studies report the laterality of the ischemic lesion and stimulation site explicitly and ideally stratify or randomize animals according to this factor to clarify the generalizability of taVNS across lesion configurations.

The clinical potential of TaVNS in treating PSCI is based on the complex pathogenesis of PSCI. Following stroke, the brain often undergoes a series of cascade reactions, including acute ischemia-induced neuronal death and subsequent secondary injury (Brown, 2021; Zeng et al., 2024). These processes dismantle functional brain networks and, through neuroinflammation, blood–brain barrier disruption, and metabolic waste accumulation, severely compromise cognitive recovery (Lu et al., 2024). Among them, choline serves as a precursor to acetylcholine, participating in cholinergic transmission and signal transduction, while reduced or deficient brain choline levels can lead to cholinergic impairment, which is closely associated with memory and attention disorders (Schliebs and Arendt, 2006; Schliebs and Arendt, 2011). Therefore, taVNS activates the cholinergic anti-inflammatory pathway via the auricular branch of the vagus nerve and may directly counteract these key pathological processes, thereby protecting cognitive function after stroke (Han et al., 2025).

Our findings demonstrate that taVNS therapy enhances neuroplasticity and effectively reduces neuronal degenerative damage. In addition, taVNS reduces astrocyte proliferation in the hippocampal and cortical regions after stroke, promotes angiogenesis, reduces blood–brain barrier (BBB) leakage in the brain, and inhibits ferroptosis-induced oxidative stress (Kuo et al., 2016; Wu et al., 2018; Sun R. et al., 2025; Choi et al., 2022; Gong et al., 2025). This finding is consistent with many previous studies, indicating that the mechanism of action of taVNS treatment after stroke primarily involves anti-inflammation, neuroplasticity, angiogenesis, and blood–brain barrier protection (Laurido-Soto et al., 2025; Sun J. et al., 2025). After stroke, the reciprocal interplay among inflammation, BBB breakdown, and neuronal loss drives secondary brain injury, including cognitive impairment (Huang et al., 2022; Lu et al., 2024). Cytokines released during neuroinflammation propagate tissue damage through pathways such as ERK/MAPK (Mao et al., 2026). Moreover, increased BBB permeability is often closely associated with neurodegeneration and inflammation-related cognitive impairment (Sweeney et al., 2019). In addition, disruption of vascular integrity and reduced CSF circulation dynamics can lead to the accumulation of metabolic waste, exacerbating post-stroke cognitive impairment (Mathias et al., 2025).

Studies have shown that the vagus nerve is extensively involved in the regulation of inflammatory injury via the cholinergic anti-inflammatory pathway. Although the specific anti-inflammatory mechanisms of taVNS are not yet fully understood, the studies we included demonstrated that it may be related to the repair of BBB tight junction proteins through the Wnt7/β-catenin signaling pathway (Sun R. et al., 2025). Inhibiting microglia and modulating the phenotypic polarization of astrocytes to promote their transformation into the neuroprotective A2 subtype, while simultaneously reducing neurotoxic A1 reactive astrocytes located at the injury center (such as the hippocampal CA1 region and cortex), is also a proposed anti-inflammatory mechanism of taVNS, supported by evidence from invasive and non-invasive vagus nerve stimulation studies (Jiang et al., 2025). In addition, taVNS promotes the release of neurotransmitters, such as Ach, and the expression of cholinergic receptors nAChR α4 and α7, thereby accelerating the functional remodeling of the central nervous system (Gong et al., 2025; Li et al., 2020).

The mechanisms by which taVNS ameliorates cognitive impairment are complex. Overall, taVNS does not act on a single pathway but instead intervenes in multiple key pathological processes involved in the development and progression of PSCI. It suppresses inflammatory responses upstream, reduces apoptosis and ferroptosis-associated oxidative stress midstream, and downstream promotes neuronal and vascular repair, protects neural signaling pathways, and decreases the accumulation of metabolic waste, thereby forming a systematic and comprehensive protective network. A central question is whether these mechanisms are directly related to the improvement of cognitive impairment by taVNS. The studies included in this meta-analysis provide various lines of evidence indicating different degrees of association between taVNS and these mechanisms, all of which contribute to improvements in cognition-related outcome measures. However, most studies had small sample sizes and substantial variations in the implementation of the interventions. Therefore, to clarify the causal relationship between cognitive improvement from taVNS treatment and the underlying mechanisms, a relatively standardized stimulation intensity, individual session duration, and total treatment course are needed. Future standardized preclinical studies should closely integrate mechanistic investigations with efficacy assessments to elucidate the detailed mechanisms by which taVNS improves cognition.

## 5 Limitations

To the best of our knowledge, this is the first systematic review and meta-analysis to quantitatively synthesize the effects of taVNS on cognitive function in PSCI animal models. By adhering to the PRISMA guidelines and preregistering the study protocol, we sought to maximize the transparency and reproducibility of this work. Methodological rigor was further reinforced through the application of the SYRCLE Risk of Bias tool, an instrument designed specifically for animal research, alongside sensitivity analyses that corroborated the stability of our principal findings. Both behavioral and neuropathological outcomes were extracted, providing a multidimensional evidence base for appraising the therapeutic benefits of taVNS.

Despite our efforts to maintain rigor throughout the analysis, this study has several notable limitations. First, the number of included studies was relatively small, and the sample sizes of each study were generally limited; therefore, Egger’s regression test for publication bias was not performed. Although the visual inspection of the funnel plot suggested approximate symmetry, this may be attributable to the small number of studies rather than the true absence of bias. While the observed effect sizes were relatively high, this may be jointly influenced by small sample sizes, genuinely strong effects, or measurement error. Further validation in studies with larger samples is warranted. Accordingly, when appraised using the GRADE framework, the certainty of evidence for the pooled outcomes was predominantly rated as low, and the observed effect sizes should therefore be interpreted with caution. Second, there was substantial variability in the stimulation parameters among the included studies, including frequency, current intensity, pulse width, duration of individual sessions, and total number of stimulations. However, due to the limited number of studies, we were unable to conduct meta-regression or subgroup analyses; thus, the dose–effect relationships could not be systematically evaluated by sensitivity analyses alone. Heterogeneity in intervention parameters may directly affect the efficacy of taVNS in improving cognitive function. Third, all the included studies exclusively used adult male rodents, with no data on females, aged animals, or other species. Moreover, most models employed were for ischemic stroke rather than hemorrhagic stroke, which limits the external validity of the findings. Finally, the included studies provided detailed reports on allocation concealment or the implementation of blinding, nor did they mention the occurrence of any adverse events, raising the possibility of implementation and reporting bias. Notwithstanding these limitations, this meta-analysis offers a systematic overview of the current preclinical evidence and serves as a valuable reference for advancing taVNS in PSCI treatment. Future investigations should adopt more rigorous methodological standards and standardized intervention protocols.

## 6 Conclusion

The findings of this meta-analysis indicate that taVNS significantly improves memory, learning ability, and neurological deficit scores in post-stroke animal models. The underlying mechanisms likely involve activation of the NNCS, which increases ACh levels and, in turn, suppresses neuroinflammation and oxidative stress, inhibits apoptosis, and promotes neovascularization. However, due to the limited number of included studies and the lack of standardized intervention protocols across studies, the optimal stimulation parameters for taVNS remain unclear. Future research should focus on establishing standardized intervention parameters, such as stimulation intensity and duration, and further elucidating its molecular mechanisms, thereby facilitating the translation of this technology into clinical applications.

## Data Availability

The original contributions presented in the study are included in the article/Supplementary material, further inquiries can be directed to the corresponding author.
